# Resveratrol Inhibits β-Amyloid-Induced Neuronal Apoptosis through Regulation of SIRT1-ROCK1 Signaling Pathway

**DOI:** 10.1371/journal.pone.0059888

**Published:** 2013-03-28

**Authors:** Xiaowen Feng, Nan Liang, Dexiao Zhu, Qing Gao, Lei Peng, Haiman Dong, Qingwei Yue, Haili Liu, Lihua Bao, Jing Zhang, Jing Hao, Yingmao Gao, Xuejie Yu, Jinhao Sun

**Affiliations:** 1 Key Laboratory of the Ministry of Education for Experimental Teratology and Department of Anatomy, School of Medicine, Shandong University, Jinan, Shandong, P. R. China; 2 Department of Neurosurgery, Provincial Hospital of Shandong University, Jinan, Shandong, P. R. China; 3 Department of Histology and Embryology, School of Medicine, Shandong University, Jinan, Shandong, P. R. China; 4 School of public Health, Shandong University, Jinan, Shandong, P. R. China; Indian Institute of Toxicology Reserach, India

## Abstract

Alzheimer’s disease (AD) is characterized by the accumulation of β-amyloid peptide (Aβ) and loss of neurons. Recently, a growing body of evidences have indicated that as a herbal compound naturally derived from grapes, resveratrol modulates the pathophysiology of AD, however, with a largely unclear mechanism. Therefore, we aimed to investigate the protection of resveratrol against the neurotoxicity of β-amyloid peptide 25–35 (Aβ_25–35_) and further explore its underlying mechanism in the present study. PC12 cells were injuried by Aβ_25–35_, and resveratrol at different concentrations was added into the culture medium. We observed that resveratrol increased cell viability through the 3-(4,5-dimethylthiazol-2-yl)-2,5-diphenyltetrazolium bromide (MTT) and lactate dehydrogenase (LDH) colorimetric assays. Flow cytometry indicated the reduction of cell apoptosis by resveratrol. Moreover, resveratrol also stabilized the intercellular Ca^2+^ homeostasis and attenuated Aβ_25–35_ neurotoxicity. Additionally, Aβ_25–35_-suppressed silent information regulator 1 (SIRT1) activity was significantly reversed by resveratrol, resulting in the downregulation of Rho-associated kinase 1 (ROCK1). Our results clearly revealed that resveratrol significantly protected PC12 cells and inhibited the β-amyloid-induced cell apoptosis through the upregulation of SIRT1. Moreover, as a downstream signal molecule, ROCK1 was negatively regulated by SIRT1. Taken together, our study demonstrated that SIRT1-ROCK1 pathway played a critical role in the pathomechanism of AD.

## Introduction

Alzheimer’s disease (AD) was firstly described by Alois Alzheimer about a century ago [1]. It is the most common neurodegenerative disease in the elderly, which eventually impairs the cognitive function of brain. The prevalence of AD exponentially increases with age. Currently, nearly one-eighth of people older than 65 years present with AD, and worldwide prevalence of the disease is expected to be close to 30 millions by 2050 [2]. The pathological features of AD include extracellular Aβ plaques and intracellular neurofibrillary tangles [3]. Aβ protein abnormally deposits in the brain, which is the typical hallmark of AD. As a toxic factor, Aβ aggregation plays a critical role in the initiation phase of AD pathogenesis [4]. Therefore, a large amount of therapeutic efforts have been focused on reducing the toxicity of Aβ protein and preventing the formation of Aβ oligomer [5].

As a natural herbal compound, resveratrol is originally found in grapes, peanuts and other plants [6]. It has long been reported that resveratrol possesses a wide range of biological activities, such as anti-oxidant, anti-inflammatory, anti-cancer and anti-aging effects in numerous organisms [7], [8]. Recently, resveratrol has also attracted the attention from neuroscientists because of its neuroprotective properties. For example, resveratrol regulates neurological disorders including strokes and Huntington’s disease [9]. Recent studies reported that resveratrol protects neurons against peroxide (H_2_O_2_), 1-methyl-4- phenylpyridine ion (MPP) and Aβ injury [10], [11], [12]. A rat model of AD suggests that resveratrol can prevent the cognitive impairment [13]. However, the neuroprotection of resveratrol against Aβ cytotoxicity, especially the underlying mechanism, remains largely unknown due to its wide pharmacological actions. Therefore, this study investigated the protective effect of resveratrol against Aβ cytotoxicity and explored the possible underlying mechanisms.

Resveratrol triggers the overexpression of SIRT1, a member of the sirtuin family [14], [15], which is a nicotinamide adenine dinucleotide (NAD)-dependent histone deacetylase and plays an essential role in regulating cellular functions, such as transcriptional silencing of telomeres and life-span extension [16], [17]. SIRT1 is also involved in calorie restriction and aging [18]. Two recent studies strongly implied that SIRT1 exerts a role in neuroprotection. Firstly, resveratrol-induced SIRT1 expression rescues the neuronal dysfunction against polyglutamines (polyQ) toxicity in Huntington’s disease [19]. Secondly, in a mouse model of slow Wallerian degeneration, resveratrol protects neurons from degeneration due to axotomy [20]. These results suggested that resveratrol is therapeutically valuable against the neurological disorder. More recently, Julien et al. [21] declared that SIRT1 may regulate the aging and metabolic processes in AD, and the loss of SIRT1 is closely associated with the Aβ accumulation and disease progression.

ROCK is serine/threonine protein kinase. There are two different diastereomers: ROCK1 and ROCK2. The former is known partly for its role in inhibiting the non-amyloidogenic, α-secretase processing of amyloid precursor protein (APP) [22]. A previous study showed that SIRT1 overexpression in primary neurons enhances cell viability and reduces Aβ secretion and ROCK1 expression, suggesting that SIRT1 enhances α-secretase-mediated non-amyloidogenic APP processing partly via ROCK1 signaling [23]. Additionally, in experiments with squirrel monkeys, calorie restriction attenuates AD type brain amyloidosis, while the protein content of SIRT1 is increased, and ROCK1 is decreased [24]. Based on these data, we hypothesized that resveratrol protected the neurons against Aβ neurotoxicity possibly through activating the SIRT1 expression, likely involving the subsequent regulation of ROCK1.

To test the above hypothesis regarding Aβ_25–35_ neurotoxicity and explore the underlying mechanism, we examined the protective effect of resveratrol on a neurotoxic cell model of Aβ_25–35_ injury using PC12 cells. MTT and LDH assays were employed to determine the cell viability; intercellular calcium ([Ca^2+^]i) level was measured using fluorescent Ca^2+^ indicator (FLuo-3/AM); and flow cytometry with Annexin V-FITC/PI double staining was used to detect the cell apoptosis. Furthermore, real time quantitative PCR and Western blotting were performed to detect the expressions of SIRT1 and ROCK1 at both the mRNA and protein levels, respectively. Finally, SIRT1 inhibitor nicotinamide and ROCK1 inhibitor Y-27632 were used to further explore the mechanisms of both proteins in the neuroprotection against neurotoxicity by resveratrol.

## Materials and Methods

### Materials and Reagents

PC12 cell line was obtained from the Cell Resource Center of Shanghai Institutes for Biological Sciences, Chinese Academy of Sciences (Shanghai, China). Aβ_25–35_, resveratrol (3,5,4′-tihydroxy-trans-stilbene), nicotinamide and dimethyl sulfoxide (DMSO) were purchased from Sigma-Aldrich Inc. (St. Louis, MO, USA). Dulbecco’s modified Eagle’s medium (DMEM) and fetal bovine serum (FBS) were provided by Hyclone Company (Logan, UT, USA).

Aβ_25–35_ was prepared as previously described [12]. Briefly, Aβ_25–35_ was dissolved in deionized distilled water and filtered through a 0.22-µm filter (Millipore, Billerica, MA, USA). Subsequently, the solution was aged by incubating at 37°C for 7 days and then stored at −20°C. Resveratrol was dissolved in DMSO at a concentration of 100 mM and stored at −20°C. The stock solution was diluted to 5 mM with serum-free DMEM before use, and the working solution was prepared with DMEM to desired concentrations.

### Establishment of Neurotoxic Cell Model with Aβ_25–35_

PC12 cell line was maintained in DMEM supplemented with 10% FBS at 37°C in a humidified atmosphere supplied with 5% CO_2_ [25]. The culture medium was refreshed on the every third day. Before experiments, cells were seeded in culture plates at a density of 2×10^4^ cells/cm^2^. After 24 h of culture, Aβ_25–35_ at different concentrations (10, 20 and 40 µM) was added into the cell culture. Cells were examined at 24 and 48 h respectively after the exposure to Aβ_25–35_.

### Protective Effect of Resveratrol on Cultured Cells

PC12 cells were cultured in 24-well plates at 37°C in the presence of 5% CO_2_ and then divided into various groups for Aβ_25–35_ treatment as follows: 1) Aβ_25–35_ injury group, in this group, Aβ_25–35_ was added into the culture medium at a final concentration of 20 µM; 2) resveratrol protection group, which consisted of four sub-groups with the addition of 2.5, 5, 10 and 20 µl of 5 mM resveratrol, respectively, into 1 ml culture medium 2 h prior to the Aβ_25–35_ injury; thus the final concentration of resveratrol was 12.5, 25, 50 and 100 µM, respectively; 3) normal control group, which was cultured without the addition of either Aβ_25–35_ or resveratrol. An equal volume of DMSO and serum-free DMEM was added into Aβ_25–35_ injury group and normal control group. The highest DMSO concentration in the culture medium was 0.1 %, which had no effect on the cell viability [26]. After 24 or 48 h of incubation, cells were collected from all groups and subjected to various examinations.

### MTT Assay

Cell viability was assessed by MTT assay according to a previous report [27]. Briefly, cells were added with MTT solution to a final concentration of 0.5 mg/ml and incubated at 37°C for 4 h. Then the medium was gently aspirated, and DMSO was added into each well to dissolve the formazan product by shaking at room temperature for 10 min. The absorbance of each sample at a wavelength of 490 nm (A490) was determined using a microplate reader (Multiskan MK3, Thermo Labsystems, Philadelphia, PA, USA). Cell viability was quantified based on the recorded A490.

### LDH Assay

To further assess the cell viability and neurotoxicity of Aβ_25–35_, LDH assay was performed with a modified method [28] using LDH kit (Jiancheng Bioengineering Institute, Nanjing, China) according to the manufacturer’s instructions. The absorbance of each sample was measured at a wavelength of 450 nm (A450) using a microplate reader (Multiskan MK3, Thermo Labsystems, Philadelphia, PA, USA). Cell viability was then quantified based on the recorded A450.

### Intracellular Ca^2+^ Measurement

Intracellular Ca^2+^ level was examined using fluorescent Ca^2+^ indicator (FLuo-3/AM) as previously described [28]. Briefly, PC12 cells were cultured in 96-well plates in the presence or absence of Aβ_25–35_ and resveratrol, respectively. After 24 h of *in vitro* culture, the culture medium was aspirated, and the cells were washed by D-Hanks buffer. Then the cells were incubated in D-Hanks buffer containing FLuo-3/AM at a final concentration of 5 µM for 45 min at 37°C. A Zeiss LSM 780 Laser Scanning Confocal Microscope (Carl Zeiss SAS, Jena, Germany) was used to detect the fluorescence intensities. The excitation was set at a wavelength of 488 nm, and the emission was recorded at 526 nm. The obtained images were analyzed by the ZEN software package of Zeiss.

### Cell Apoptosis Analysis

PC12 cells in different groups were double-stained with Hoechst 33342 and propidium iodide (PI) at a concentration of 10 µg/ml for 15 min. Labeled cells were observed under a LSM 780 Laser Scanning Confocal Microscope (Carl Zeiss SAS, Jena, Germany). In order to further discriminate the early and late cell apoptosis and perform a quantitative analysis, flow cytometry with Annexin V-FITC/PI double staining was employed as previously described [29]. Briefly, PC12 cells were detached with 0.125% trypsin, centrifuged at 1,000 rpm for 5 min, and then washed twice with PBS. Subsequently, 5×10^5^ cells were resuspended in binding buffer and stained with Annexin V-FITC and PI for 15 min in the dark at room temperature. Finally, the fluorescence of each group was analyzed by flow cytometry (Becton Dickinson, Franklin Lakes, NJ, USA).

### Real Time Quantitative PCR

In order to investigate the protective mechanism of resveratrol, the expression of SIRT1 and ROCK1 was examined by real time quantitative PCR. Total RNA was extracted from PC12 cells using Trizol reagent (Sangon Biotech Co., Ltd., Shanghai, China). The RNA integrity was spectrophotometrically examined according to its A260/A280 absorption. Subsequently, cDNA was synthesized through reverse transcription. Real time quantitative PCR was performed on Mastercycler ep realplex (Eppendorf, Hamburg, Germany). Briefly, the amplification reaction was carried out with 40 cycles at a melting temperature of 94°C for 15 sec, an annealing temperature of 60°C for 1 min, and an extension temperature of 72°C for 50 sec. Housekeeping gene GAPDH was selected as an internal control. Following primers were used in the amplification: SIRT1, forward primer: 5′-CCAGAAACAATTCCTCCACCT-3′, reverse primer: 5′-CAGCAAGGCGAGCATAAATAC-3′; ROCK1, forward primer: 5′-ATCCACCAGGAAGGTTTATGC-3′, reverse primer: 5′-AGGCACATCGTAGTTGCTCAT-3′; GAPDH, forward primer: 5′-TGGAGTCTACTGGCGTCTT-3′, reverse primer: 5′-TGTCATATTTCTCGTGGTTCA-3′. The relative expression of the target gene at the mRNA level was determined by the 2^−ΔΔCt^ method.

### Western Blotting

PC12 cells were collected and lysed by RIPA lysis buffer (Beyotime Institute of Biotechnology, Shanghai, China). Total proteins were extracted, and the protein concentration was determined using BCA kit (Boster Biological Technology, Wuhan, China). The protein samples were separated on 8% polyacrylamide gels and electro-transferred onto nitrocellulose (NC) membranes in an ice-water environment. Blots were blocked by 5% defatted milk in Tris-buffer containing 0.1% Tween-20 and then incubated with primary anti-SIRT1 (1∶1,000, Santa Cruz Biotechnology, Inc., CA, USA) and anti-ROCK1 (1∶3,000, Sigma-Aldrich, St. Louis, MO, USA) antibodies at 4°C overnight. Subsequently, the blots were incubated with the anti-rabbit secondary antibody (1∶1,000, ZSGB-BIO ORIGENE, Beijing, China) at room temperature for 1 h. Then the blots were developed with ECL reagent (Millipore Corporation, Billerica, MA, USA) and analyzed with Quantity One software (Image J, USA).

### Effect of Nicotinamide on Resveratrol’s Protection

The role of SIRT1 in the neuroprotection of resveratrol against Aβ_25–35_ toxicity was investigated by using SIRT1 inhibitor nicotinamide. Nicotinamide was diluted in deionized distilled water and stored at 4°C. In the resveratrol protection group, nicotinamide and resveratrol were simultaneously added into the culture medium, and the final concentration of nicotinamide was 5 mM as previously reported [30]. The cell growth determined by MTT assay was used to analyze the role of SIRT1 in the protection process.

### Effects of Nicotinamide and Y-27632 on SIRT1 and ROCK1 Expression

Y-27632 is a potent and specific inhibitor of ROCK1. Nicotinamide and Y-27632 were used to further investigate whether SIRT1 regulated the ROCK1 expression. After 24 h of *in vitro* culture, PC12 cells were treated with 5 mM nicotinamide and 10 µM Y-27632, respectively. The concentration of Y-27632 was referred a previous report [31]. Cells without the addition of either nicotinamide or Y-27632 were served as the normal control group. After 24 h treatment with the inhibitors, the expressions of SIRT1 and ROCK1 were analyzed by real time quantitative PCR and Western blotting.

### Statistical Analysis

Statistical analysis was performed using the SPSS 17.0 software. Data of MTT and LDH assays were processed with analysis of variance (ANOVA) followed by Newman-Keuls. Other data were analyzed by Student’s *t*-test. All the data were expressed as mean ± S.E.M. *p* < 0.05 was considered as statistically significant.

## Results

### Resveratrol Increases Cell Viability Against Aβ_25–35_

**Figure 1:**
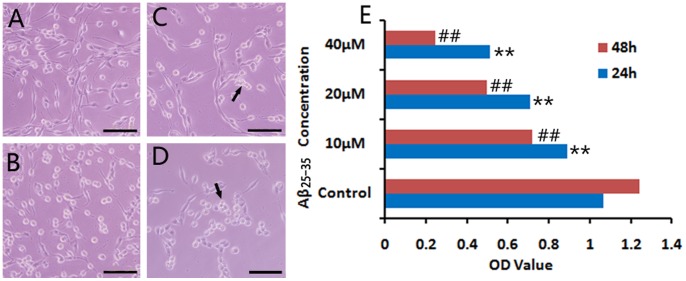
Survival of PC12 cells exposed to Aβ_25–35_. PC12 cells were cultured for 24 and 48 h with 10, 20 and 40 µM Aβ_25–35_, respectively. After 24 h *in vitro* culture, (A) PC12 cells of the normal control group grew well with long neurites. (B) While incubation with 10 µM Aβ_25–35_, neurites of cells retracted gradually. (C) When added 20 µM Aβ_25–35_ in the medium, the damage to cells was obvious, and some cell debris appeared. (D) The 40 µM Aβ_25–35_ strongly insulted cells resulting in loss of more cells. After 48 h with Aβ_25–35_, more cells floated in the medium, and cell debris increased. Arrows indicated cell debris. Cell viability was assessed by MTT assay. Absorbance values were presented as mean ± S.E.M. **p < 0.01, compared with the normal control group at 24 h in culture; ^##^ *p* < 0.01, compared with the normal control group at 48 h in culture. Data were processed by two-way ANOVA followed by Newman-Keuls test (n = 9). Scale bars: A-D, 100 µm.

As shown in Fig. 1A, PC12 cells grew with long neurite in DMEM; when exposed to Aβ_25–35_, the damage to cells was evident under microscope. The neurites slowly disappeared, and the network was collapsed. Aβ_25–35_ was added into the medium at three doses (10, 20 and 40 µM), and its cytotoxic effect became visible after the exposure to 20 µM Aβ_25–35_ for 24 h, as evidenced by retracted neurites and some cell debris (Fig. 1C). While 10 µM Aβ_25–35_ slightly damaged the cells (Fig. 1B), and PC12 cells were strongly insulted by 40 µM Aβ_25–35_ with severe cell loss (Fig. 1D). After exposed to Aβ_25–35_ for 48 h, more cells floated in medium, and the cell debris increased. In addition, MTT assay also confirmed the negative correlation between the cell viability and Aβ_25–35_ concentrations (Fig. 1E). Therefore, Aβ_25–35_ of 20 µM was selected to further assess the protective mechanism of resveratrol.

**Figure 2:**
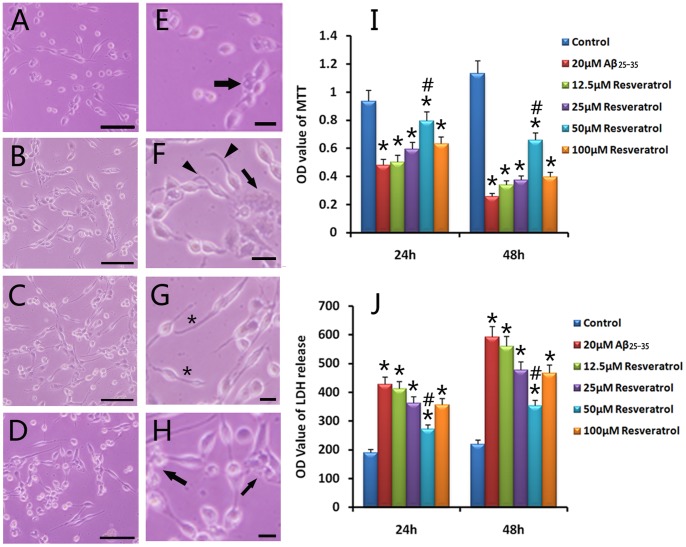
Protection of resveratrol on PC12 cells against Aβ_25–35_. PC12 cells were cultured in DMEM medium added with 12.5 (A), 25 (B), 50 (C) and 100 µM (D) resveratrol 2 h prior to the addition of 20 µM Aβ_25–35_. E-H are the magnification of A-D, respectively. (A) In protection group of 12.5 µM resveratrol, many cells floated and disappeared. In 25 (B) and 100 (D) µM resveratrol groups, although cells grew with neurites, some cells were swollen and in a round shape. However, 50 µM (C) resveratrol promoted the neurite growth, with fewer cell debris. Arrows (E, F and H) indicated cell debris. Asterisks (G) indicated neurites. Arrowheads (F) indicated curved neurites. Cell viability was further determined by MTT (I) and LDH (J) assays. Absorbance values were recorded as mean ± S.E.M. *p < 0.01, compared with the Aβ_25–35_ injury group; ^#^ *p* < 0.01, compared with values of 12.5, 25 and 100 µM resveratrol in 24 h and 48 h groups, respectively. Data were processed by two-way ANOVA followed by Newman-Keuls test (n = 9). Scale bars: A–D, 100 µm; E, 30 µm; F, 30 µm; G, 15 µm; and H, 20 µm.

Resveratrol at 12.5, 25, 50 and 100 µM was added to PC12 cells 2 h prior to the addition of 20 µM Aβ_25–35_, and the protective effect was compared among groups after 24 h of treatment (Fig. 2A–D, all *p* < 0.01). In the group with 50 µM resveratrol, PC12 cells showed normal growth with neurites, and only little cell debris appeared (Fig. 2G). 12.5 µM resveratrol exhibited a weak protective effect (Fig. 2E). Resveratrol at 25 and 100 µM exerted a similar protective effect; however, some cells swelled and turned into a round shape (Fig. 2F and H). The protective effect of resveratrol was further confirmed using MTT and LDH assays (Fig. 2I and J). In MTT assay, the A490 values in resveratrol protection groups were all higher than those in the Aβ_25–35_ injury group (all *p* < 0.01), though they were still lower than those in the normal control group (*p* < 0.01). LDH assays were also carried out to further verify the change in cell viability, and the results demonstrated that resveratrol decreased the LDH level and attenuated the Aβ_25–35_-induced cytotoxicity.

### Resveratrol Reduces Aβ_25–35_-induced Intracellular Ca^2+^ Level

**Figure 3:**
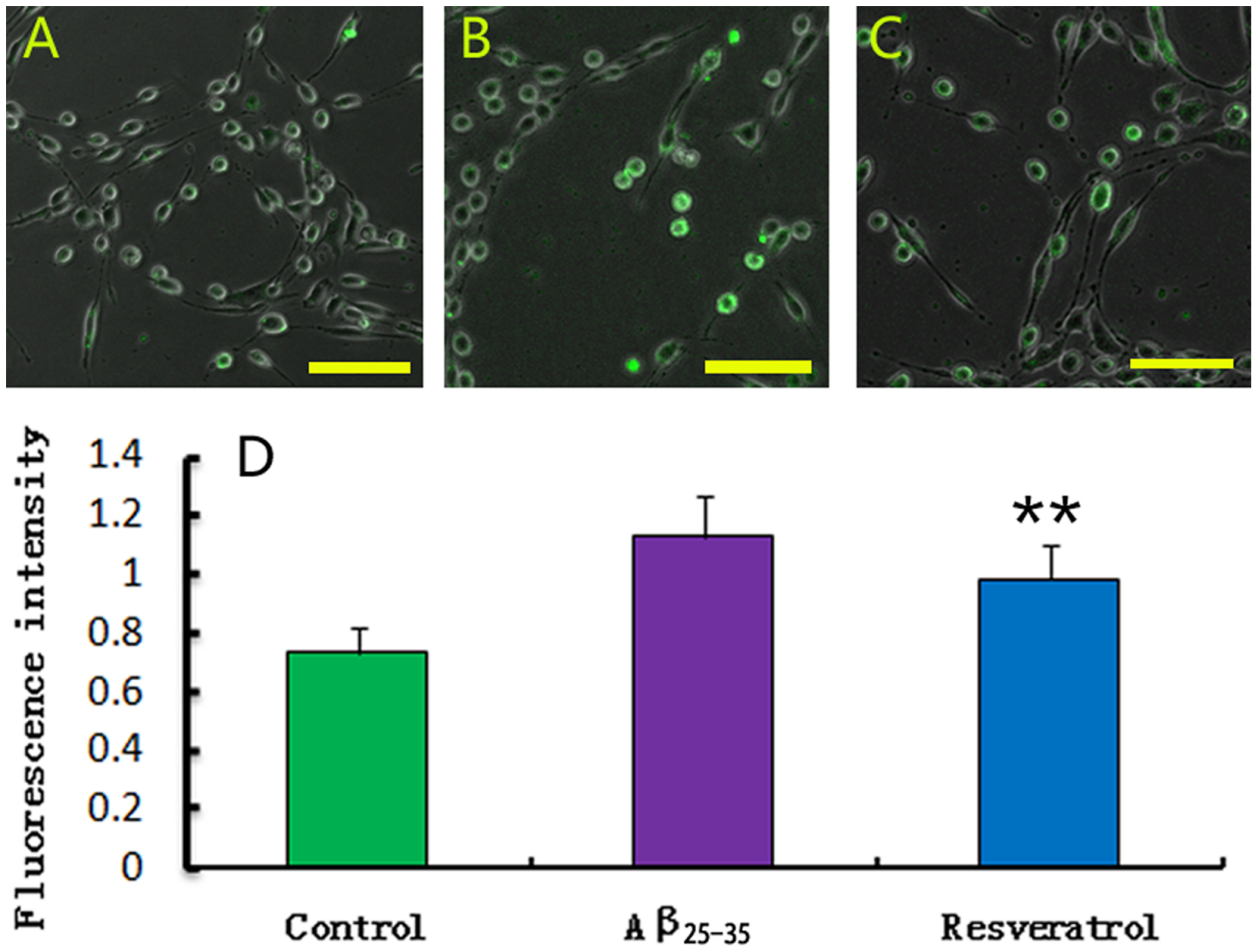
Change of intracellular Ca^2+^ level. Fluorescent Ca^2+^ indicator fluo-3/AM was used to determine the [Ca^2+^]i level. Only a few PC12 cells loaded Ca^2+^ indicator with green fluorescence in the normal control group (A). After treated with 20 µM Aβ_25–35_ for 24 h *in vitro*, many cells were bright green with high fluorescence intensity (B). When added with 50 µM resveratrol, PC12 cells loaded with Ca^2+^ indicator decreased (C). Statistical analysis showed that the fluorescence intensity in the resveratrol protection group was significantly lower than that in the Aβ_25–35_ injury group, though it was still higher than that in the normal control group (D). Values of fluorescence intensity were presented as mean ± S.E.M. **p < 0.01, compared with the Aβ_25–35_ injury group by student’s *t*-test (n = 9). Pictures of A–C were captured with Zeiss LSM 780 laser cofocal microscope at 200× magnification. Scale bars: A–C,100 µm.

The intracellular Ca^2+^ level was determined using the fluorescent Ca^2+^ indicator, Fluo-3/AM. The lowest [Ca^2+^]i level was detected from the normal control group; only a few PC12 cells exhibited weak green fluorescence (Fig. 3A). Interestingly, the [Ca^2+^]i level was increased when exposed to Aβ_25–35_ for 24 h, with many cells showing high level of green fluorescence intensity (Fig. 3B). In the presence of resveratrol, the [Ca^2+^]i level was significantly decreased after 24 h (Fig. 3C). However, resveratrol did not bring the [Ca^2+^]i back to its original level. Statistical analysis showed that the [Ca^2+^]i level in Aβ_25–35_ injury group was significantly different from that of the resveratrol (50 µM) protection group (Fig. 3D, *p* < 0.01). Therefore, resveratrol decreased the [Ca^2+^]i level induced by cytotoxic Aβ_25–35_.

### Resveratrol Prevents Cell Apoptosis Caused by Aβ_25–35_

**Figure 4:**
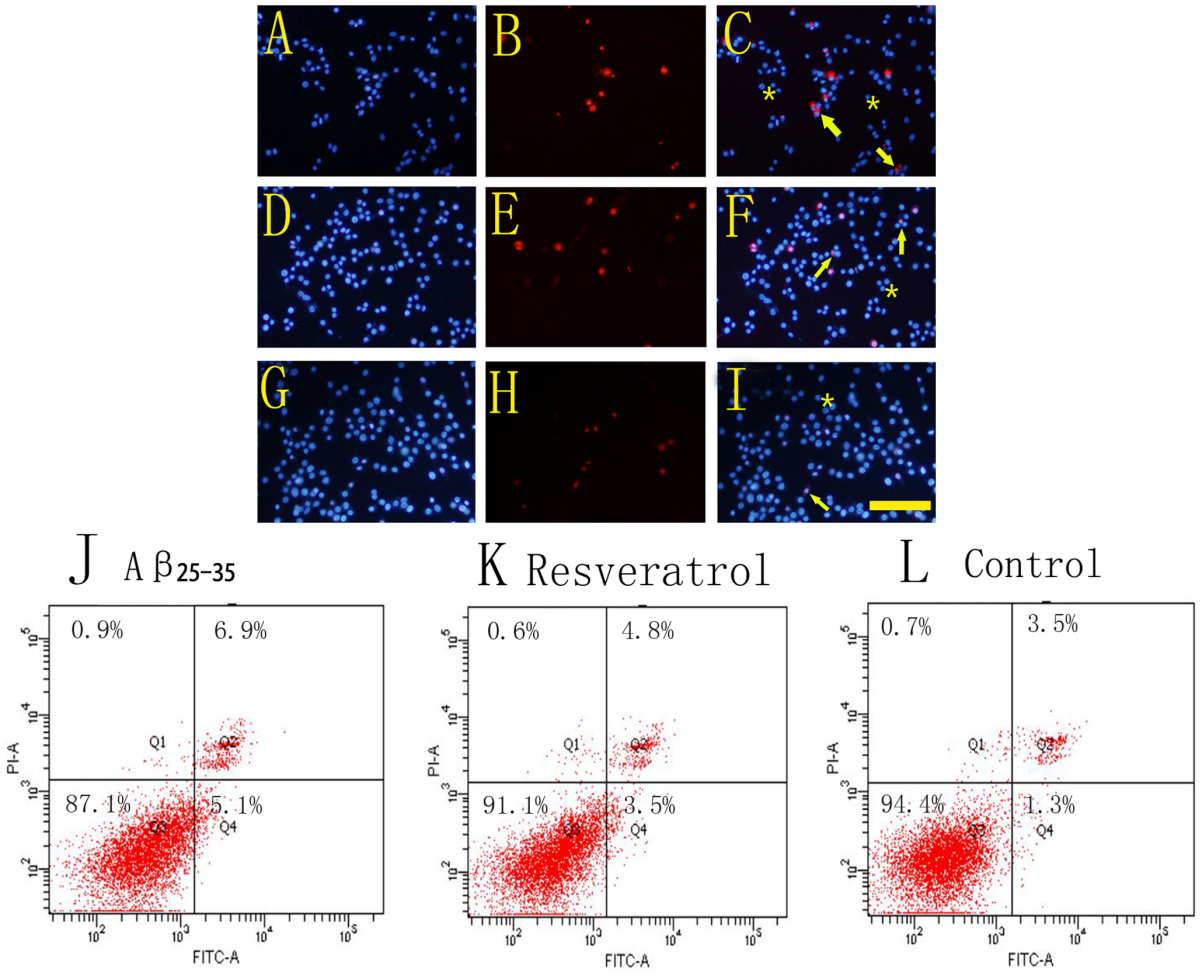
Effects of resveratrol on Aβ_25–35_-induced cell apoptosis. PC12 cells of Aβ_25–35_ injury group (A–C), resveratrol protection group (D–F) and the normal control group (G–I) were all stained with Hoechst 33342 (blue) and PI (red). C, F and I were the merge pictures of the corresponding stainings. Under fluorescence microscope, the nuclei of apoptosis cells were stained with bright red fragmented nuclei containing one or more lobes of condensed chromatin (Arrows in C, F and I). While the survival cells were stained with bright blue fluorescence (Asterisks in C, F and I). J, K and L were the results of flow cytometry of 30 µM Aβ_25–35_, 50 µM resveratrol, and the normal control group, respectively. Lower left quadrant area indicated the survival cells. The early and late apoptosis cells were presented by the lower right and upper right quadrants, respectively. Statistical analysis indicated that resveratrol significantly reduced both early and late cell apoptosis, compared with the Aβ_25–35_ injury group by students’s *t*-test (*p* < 0.01, n = 6). Pictures of A–I were captured with Zeiss LSM 780 laser cofocal microscope at 200× magnification. Scale bars: A–I, 100 µm.

Through double staining of Hoechst 33342 and PI, the apoptotic cells exhibited the fragmented nuclei in bright red, whereas the survived cells displayed the intact nuclei in bright blue. In the Aβ_25–35_ injury group, many apoptotic nuclei in red were observed (Fig. 4A–C). However, the number of apoptotic nuclei was decreased in the resveratrol protection group (Fig. 4D–F). Few apoptotic cells were visible in the normal control group (Fig. 4G–I). To further confirm above results, we evaluated the protective effect of resveratrol on apoptosis of PC12 cells using flow cytometry with Annexin V-FITC/PI double staining, which can also distinguish the early and late apoptosis. When exposed to 20 µM Aβ_25–35_, slight apoptosis was induced in PC12 cells compared with the normal control group (*p* < 0.01). Resveratrol at 50 µM reduced the cell apoptosis compared with that in the Aβ_25–35_ injury group, and the early apoptosis obviously decreased (*p* < 0.01). To confirm resveratrol’s protection on cultured cells, a higher dose of 30 µM Aβ_25–35_ was also used in this study, and the results showed that 30 µM Aβ_25–35_ induced evident cell apoptosis around 11.9% compared with the normal control group (Fig. 4J and L, *p* < 0.01), and resveratrol significantly inhibited apoptotic cells to 8.4% compared with the Aβ_25–35_ injury (Fig. 4J and K, *p* < 0.01). Both early and late apoptosis were inhibited by 50 µM resveratrol.

### Resveratrol Upregulates the SIRT1 Expression and Downregulates the ROCK1 Expression in Aβ_25–35_-treated Cells

**Figure 5:**
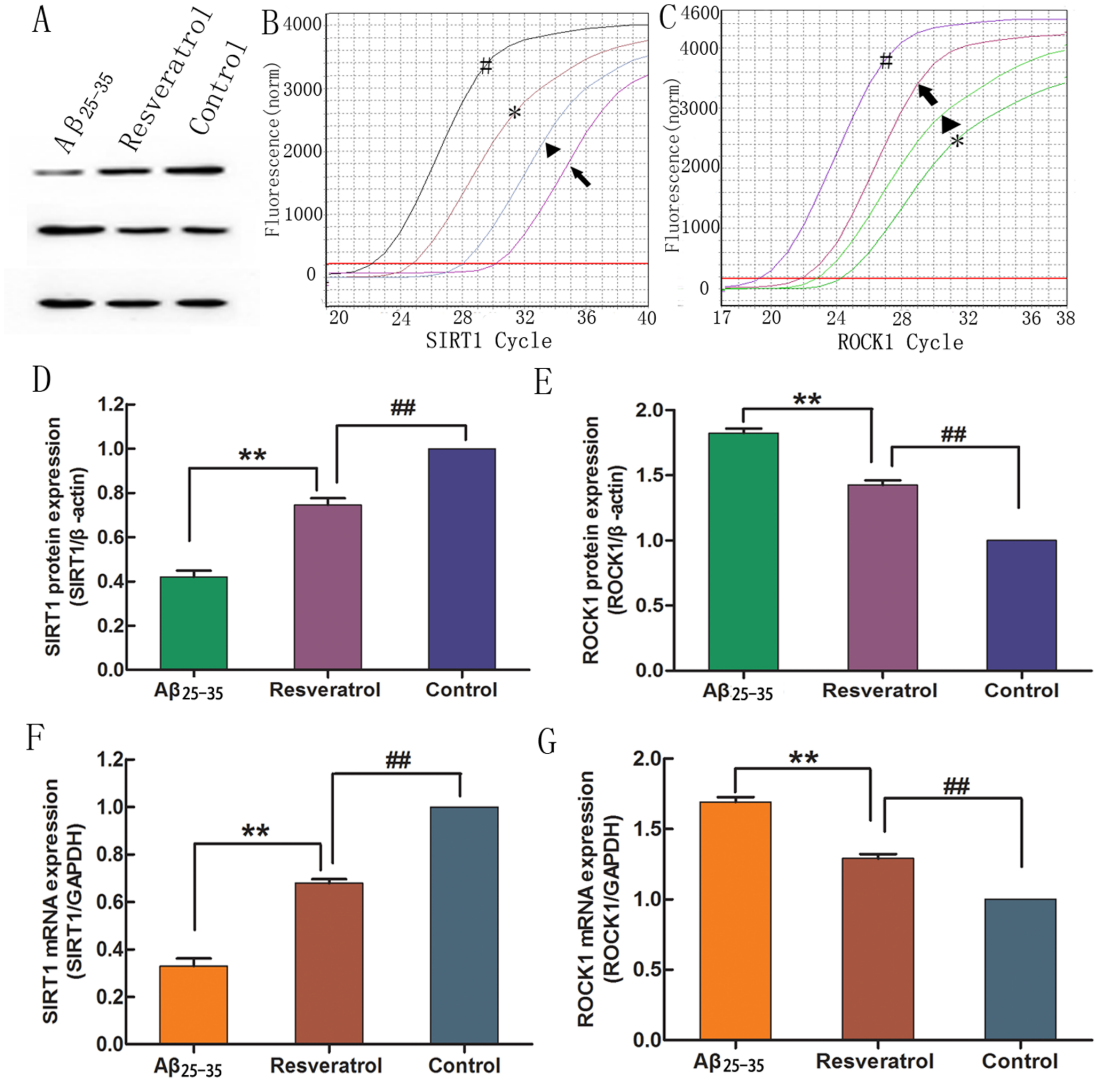
Resveratrol rescued SIRT1 and ROCK1 expression. SIRT1 and ROCK1 expressions were determined by Western blotting (A) and real time quantitative PCR (B and C). The 20 µM Aβ_25–35_ significantly decreased protein (D) and mRNA (F) expression of SIRT1, but markedly increased protein (E) and mRNA (G) expression of ROCK1. With 50 µM resveratrol in culture medium, SIRT1 and ROCK1 expressions were partly recovered. In real time quantitative PCR (B and C), pound presented GAPDH; asterisk presented the normal control group; arrowhead presented the resveratrol protection group; and arrow presented the Aβ_25–35_ injury group. **p < 0.01, compared with the Aβ_25–35_ injury group; ^##^ *p* < 0.01, compared with the normal control group by student’s *t*-test (n = 3).

To further explore the protective mechanism of resveratrol on PC12 cells against Aβ_25–35_ neurotoxicity, we examined the expression of SIRT1 and ROCK1 at the mRNA level using real time quantitative PCR. As shown in Fig. 5B, the SIRT1 expression was significantly decreased for around 8-fold by Aβ_25–35_ compared with the normal control group (Fig. 5F, *p* < 0.01). Meanwhile, the expression of SIRT1 was markedly increased when PC12 cells were incubated with resveratrol. However, the ROCK1 expression was increased by Aβ_25–35_ compared with the normal control group, while such increase was significantly attenuated by resveratrol (Fig. 5C and G, *p* < 0.01). We also analyzed the protein expression of SIRT1 and ROCK1 by Western blotting. The SIRT1 expression was increased in the presence of resveratrol compared with that in the Aβ_25–35_ injury group (Fig. 5A and D, *p* < 0.01), though it remained lower than that in the normal control group (Fig. 5D, *p* < 0.01). The ROCK1 expression was decreased in the presence of resveratrol compared with that in the Aβ_25–35_ injury group (Fig. 5A and E, *p* < 0.01); however, it was still higher than that in the normal control group (Fig. 5E, *p* < 0.01). These data implied that SIRT1 and ROCK1 partially participated in the neuroprotection of resveratrol against Aβ_25–35_ injury.

### Nicotinamide Partially Attenuates the Neuroprotective Effect of Resveratrol on PC12 Cells

**Figure 6:**
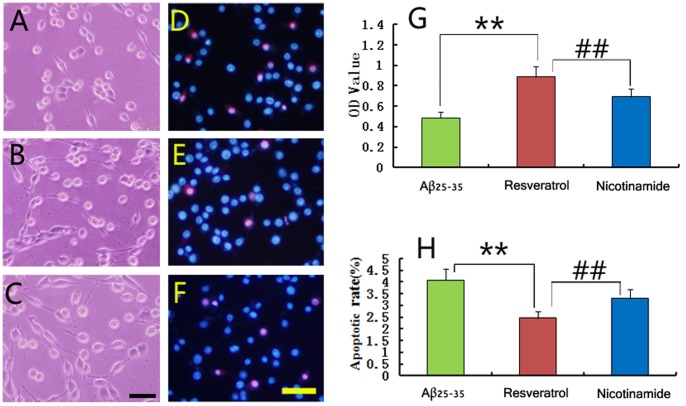
SIRT1 inhibitor nicotinamide partly attenuated resveratrol’s protection against Aβ_25–35_. To further determine the role of SIRT1 in resveratrol’s protection of PC12 cells, nicotinamide was added into culture medium in the resveratrol protection group. The growth of PC12 cells was observed under phase contrast microscope (A–C), and cell apoptosis was detected by Hoechst 33342 (blue) and PI (red) double staining (D–F) and observed under Zeiss LSM 780 laser cofocal microscope. Nicotinamide (C and F) attenuated the protection of resveratrol (B and E) on PC12 cells. A and D were the Aβ_25–35_ injury group. MTT assay (G) and cell apoptosis analysis (H) were preformed. Data were presented as mean ± S.E.M. **p < 0.01, compared with the Aβ_25–35_ injury group; ^##^ *p* < 0.01, compared with the nicotinamide group by Student’s *t*-test (n = 9). Scale bars: A-C, 50 µm; D-F, 50 µm.

Based on the changes of SIRT1 expression in PC12 cells, we investigated the role of SIRT1 in the neuroprotection of resveratrol by adding nicotinamide, the SIRT1 inhibitor, into the culture medium of the resveratrol protection group. The results showed that although the growth of PC12 cells in the resveratrol protection group (Fig. 6B) was significantly improved compared with that in the Aβ_25–35_ injury group (Fig. 6A), such protective effect of resveratrol was attenuated by the addition of nicotinamide, evidenced by loss of neurites in more cells (Fig. 6C). Moreover, Hoechst 33342/PI double staining showed that more apoptotic cells were observed with nicotinamide treatment (Fig. 6D–F). MTT assay also confirmed that the cell viability in the nicotinamide group was lower than that in the resveratrol protection group (Fig. 6G, *p* < 0.01), though it remained higher than that in the Aβ_25–35_ injury group. Furthermore, cell apoptosis analysis demonstrated that the apoptotic rate in the nicotinamide group was higher than that in the resveratrol protection group (Fig. 6H, *p* < 0.01). Therefore, resveratrol protected PC12 cells against the Aβ_25–35_ neurotoxicity partially through regulating the SIRT1 expression.

### SIRT1 Downregulates ROCK1 Expression

**Figure 7:**
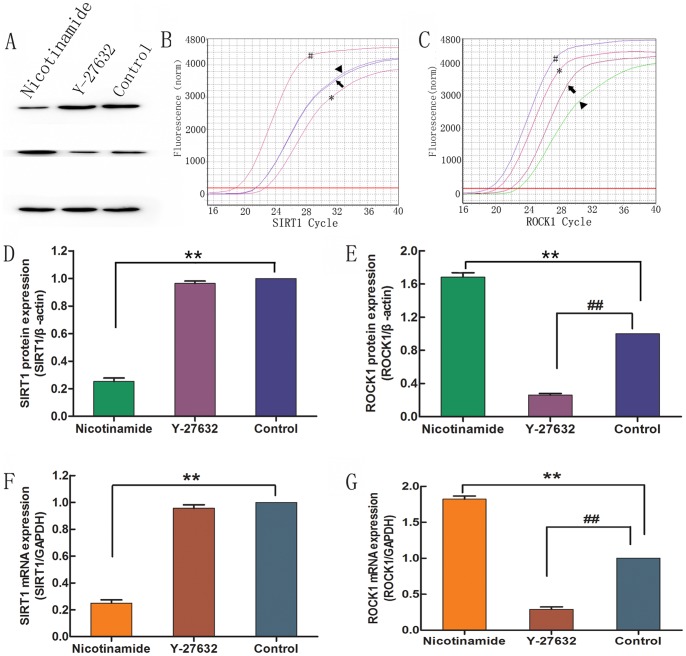
Nicotinamide inhibited SIRT1 expression and activated ROCK1 expression. SIRT1 and ROCK1 expressions were determined by Western blotting (A) and real time quantitative PCR (B and C). Nicotinamide significantly inhibited the protein (D) and mRNA (F) expression of SIRT1 but increased the protein (E) and mRNA (G) expressions of ROCK1. Y-27632 significantly inhibited the protein (D) and mRNA (F) expressions of ROCK1 but did not influence the protein (E) and mRNA (G) expression of SIRT1. In real time quantitative PCR (B and C), pound presented GAPDH*;* asterisk presented the nicotinamide group; arrowhead presented the Y-27632 group; and arrow presented the normal control group. **p < 0.01 and ^##^ *p* < 0.01, both compared with the normal control group by student’s *t*-test (n = 3).

To further explore the mechanism in the neuroprotective effects of resveratrol against Aβ_25–35_ and whether SIRT1 regulated the ROCK1 expression, the expression of SIRT1 and ROCK1 were determined at the mRNA level in PC12 cells treated with nicotinamide or Y-27632 (a selective inhibitor of ROCK1). The results showed that nicotinamide significantly inhibited the SIRT1 expression and increased the ROCK1 expression compared with the normal control group (Fig. 7B, C, F, and G, *p* < 0.01). Y-27632 greatly inhibited the ROCK1 expression compared with the normal control group (Fig. 7C and G, *p* < 0.01); however, the expression of SIRT1 was not affected. Furthermore, we analyzed the protein expression of SIRT1 and ROCK1 by Western blotting. The expression of SIRT1 and ROCK1 in groups treated with nicotinamide or Y-27632 was consistent with the results of real time quantitative PCR (Fig. 5A, D, and E, *p* < 0.01). Therefore, nicotinamide inhibited the SIRT1 expression and activated the ROCK1 expression, whereas Y-27632 only inhibited the ROCK1 expression. These data indicated that ROCK1 was a downstream signal molecule and could be suppressed by SIRT1.

## Discussion

In the present study, we established an *in vitro* damage model of PC12 cells using Aβ_25–35_ and provided new evidences on the protective effect of resveratrol against Aβ neurotoxicity. Resveratrol increased the cell viability, and attenuated the intracellular Ca^2+^ level and Aβ_25–35_-induced cell apoptosis. In particular, we explored the underlying mechanism and found that resveratrol recovered the Aβ_25–35_-suppressed SIRT expression. Therefore, the neuroprotection of resveratrol against Aβ_25–35_ in PC12 cells was partially mediated by upregulating the SIRT1 expression, which in turn negatively regulated the ROCK1 activity.

Resveratrol is naturally synthesized when plant suffers from fungal attack and is exposed to ultraviolet light, and it is mainly distributed in the skin and seeds of purple grapes and peanuts [32]. As an active ingredient of polyphenols in red wine and many plants, resveratrol has received increasing attention due to its therapeutic potentials in treating inflammation, cancer and neurologic disorders. Actually, quite a few reports suggested that drinking red wine can attenuate the cognitive degeneration, which is mainly attributed to resveratrol, the polyphenol compound [33], [34]. This study showed that resveratrol increased the cell viability against Aβ_25–35_ toxicity. The growth of PC12 cells in the resveratrol protection group was significantly improved compared with that in the Aβ_25–35_ injury group. Besides resveratrol, other herbal medicines, such as Ginkgolide B [35] and Bacopa monnieri [36], also demonstrate neuronal protection effects against Aβ_25–35_. In recent years, more than 50 different plants or herbs, either in pure molecular form or in specific extracts, have been identified potentially useful for AD treatment [37]. Investigation of the health benefits of these natural compounds including resveratrol poses substantial challenges to modern medicine; especially, herb-derived drugs become popular in recent days because of their good safety profiles and low incidence of side effects [38].

In our study, we found that the protection of 50 µM resveratrol was better than that in other groups. Conte et al. [39] also suggested that 50 µM resveratrol protects PC12 cells from Aβ_1–41_ injury, and the concentration higher than 50 µM exerts the inhibitory effect, which is consistent with our study. However, Alvira et al. [40] reported that 100 µM resveratrol still renders the protection on cerebellar neurons against MPP, which is slightly better than 50 µM. This is possibly because that Aβ_25–35_ and Aβ_1–41_ might insult cells differentially, and the resistance to drugs of different cells also has variations among studies.

Annexin V-FITC/PI double staining can sensitively identify the apoptotic cells, including both early- and late-apoptotic cells, which can give a quantitative analysis. By flow cytometry analysis, we demonstrated that resveratrol inhibited the cell apoptosis induced by Aβ_25–35_. In the presence of 50 µM resveratrol, early apoptosis induced by slight Aβ_25–35_ injury was significantly decreased, and both early and late apoptosis were reduced treated with Aβ_25–35_ at higher concentration.

A growing body of evidences demonstrate that [Ca^2+^]i is a universal signaling molecule that regulates many cellular functions, and it is one of the key elements of apoptotic signaling pathways [41], [42]. Many toxic factors trigger apoptosis by early transient elevation of intracellular free calcium, thereby resulting in the increase of membrane permeability and mitochondrial membrane disruption [43]. Ferreiro et al. [44] reported that Aβ_25–35_ changes the [Ca^2+^]i homeostasis. Therefore, we measured the changes of [Ca^2+^]i level in the prevention of resveratrol against Aβ_25–35_ toxicity. Our data indicated that Aβ_25–35_ obviously increased the [Ca^2+^]i levels in PC12 cells, while resveratrol restored the Ca^2+^ homeostasis. This was consistent with the high signals in MTT and LDH assays. Therefore, the increase of [Ca^2+^]i was the initial step in the injury of PC12 cells, and resveratrol possibly protected cells from the damage of Aβ_25–35_ toxicity at the beginning. Further study is necessary to determine the effect of resveratrol on intracellular Ca^2+^ homeostasis.

Resveratrol is also a calorie restriction mimetics that triggers the overexpression of sirtuins, of which SIRT1 is closely associated with aging and Aβ accumulation [14], [21]. To further explore the underlying mechanisms of resveratrol’s protection against Aβ_25–35_ neurotoxicity, we examined the expression of SIRT1, a silent information regulator. Our results showed that Aβ_25–35_ obviously reduced the expression of SIRT1, which was partially recovered by resveratrol. A current report also suggested the low expression of SIRT1 in cerebral cortex of AD [15].

Recently, Qin et al. [23] reported that α-secretase activity is increased in SIRT1 transgenic mice, which is correlated with a reduction in the ROCK1 expression. Coincidentally, a similar result has been reported in a squirrel monkey model of calorie restriction [24]. In this study, Aβ caused the loss of SIRT1 and the increase of ROCK1. Importantly, resveratrol increased the SIRT1 level accompanied by ROCK1 reduction, suggesting that SIRT1 and ROCK1 played key roles in the anti-neurotoxicity of resveratrol. However, the feedback loop between SIRT1 and ROCK1 remains unclear. We revealed the regulation of ROCK1 by SIRT1 using nicotinamide and Y-27632, which are inhibitors of SIRT1 and ROCK1, respectively. As expected, nicotinamide significantly inhibited the SIRT1 expression and simultaneously increased the ROCK1 expression. Y-27632 greatly inhibited the ROCK1 expression, while had no effect on the SIRT1 expression. These results indicated that ROCK1 was a downstream signal molecule and downregulated by SIRT1, which in turn inhibited the Aβ_25–35_-induced cell apoptosis.

Although we demonstrated the connection among resveratrol, SIRT1, ROCK1 and Aβ in the present study, the downstream events in neuronal protection after the regulation of SIRT1 and ROCK1 expression remain unclear. Recent reports demonstrated that SIRT1 also regulates two apoptosis-associated proteins, P53 and FOXO [45], [46]. Additionally, a recent study indicated that SIRT1 inhibits the nuclear factor kappa B signaling and protects the neurons [47]. Moreover, it has also been shown that resveratrol directly binds to Aβ_42_ and interferes with its aggregation, leading to an attenuated Aβ cytotoxicity [48]. More likely, these functions play a synergistic role in the neuronal protection, which warrants further investigations.

Taken together, resveratrol protected PC12 cells from Aβ-induced neurotoxicity and inhibited the cell apoptosis. It prevented the LDH leakage and maintained the intracellular Ca^2+^ homeostasis. Especially, Aβ_25–35_ suppressed the SIRT1 expression and hence upregulated the expression of downstream ROCK1, which was significantly recovered by resveratrol. Our data further demonstrated that the anti-apoptosis effects of resveratrol were partially attributed to the SIRT1-ROCK1 pathway. This study provided new insights into the pathogenesis and treatment of neurodegenerative disease.
